# Supplementary material for: Characterization of a New Glucose-Tolerant GH1 β-Glycosidase from Aspergillus fumigatus with Transglycosylation Activity
Source: Int J Mol Sci. 2023 Feb 24;24(5):4489. doi: 10.3390/ijms24054489 (PMC10003650; doi:10.3390/ijms24054489)
Supplement: Supplementary file 1 [file ijms-24-04489-s001.zip › ijms-2149764-supplementary.pdf]

Characterization of a new glucose-tolerant GH1 β-glycosidase from *Aspergillus fumigatus* with transglycosylation activity

Lucas Matheus Soares Pereira[1], Aline Vianna Bernardi[1], Luis Eduardo Gerolamo[1], Wellington Ramos Pedersoli[2], Cláudia Batista Carraro[2], Roberto do Nascimento Silva[2], Sergio Akira Uyemura[3] and Taísa Magnani Dinamarco[1,3*]

[1]Faculty of Philosophy, Sciences and Literature of Ribeirão Preto, Chemistry Department, University of São Paulo, Ribeirão Preto, São Paulo, Brazil. [2]Ribeirão Preto Medical School, Department of Biochemistry and Immunology, University of São Paulo, Ribeirão Preto, São Paulo, Brazil, [3]Faculty of Pharmaceutical Science, Department of Clinical, Toxicological and Bromatological Analysis, University of São Paulo, Ribeirão Preto, São Paulo, Brazil.

*****Correspondence: author: tdinamarco@ffclrp.usp.br; Tel.: +55-16-3315-9121; Fax: +55-16-3315-9101

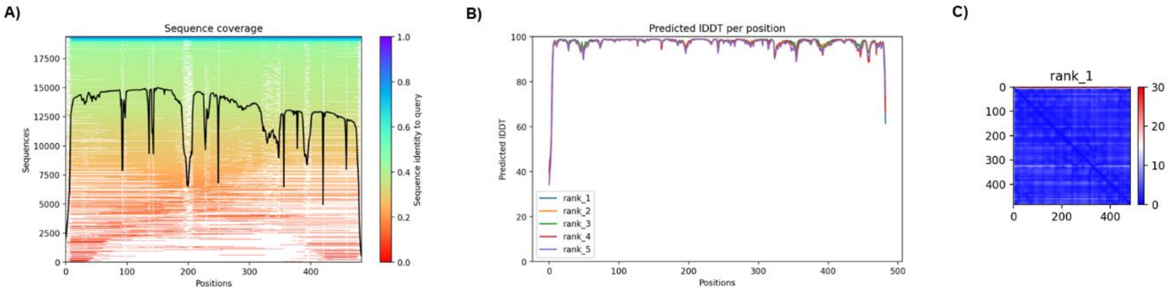

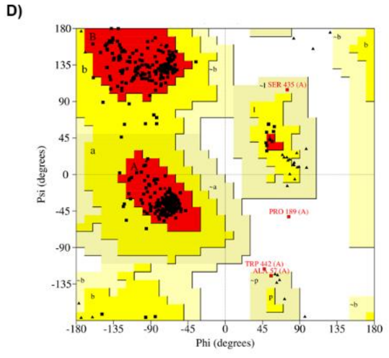

E)

PROCHECK summary

Residues in most favored regions (A, B, L)	382	93.2%
Residues in additional allowed regions (a, b, l, p)	25	6.1%
Residues in generously allowed regions (~a,~b,~l,~p)	2	0.5%
Residues in disallowed regions	1	0.2%
Number of non-glycine and non-proline residues	410	100%
Number of end-residues (excl. Gly and Pro)	2	
Number of glycine residues (shown in triangles)	42	
Number of proline residues	29	
Total number of residues	483	

Figure S1: Prediction and validation of AfBgl1.3 tridimensional structure. A) Sequence coverage from the alignments made for structure prediction. **B)** pLDDT for each model predicted, showing the confidence metric per residue. For the model used in this study, confidence values were pLDDT = 97.4, pTM=0.953. **C)** PAE (Predicted Alignment Error) plot for the model used in the study, showing the high model quality. **D)** Ramachandran plot and **(E)** PROCHECK summary. It

is possible to notice that 93.2% of residues are located in the most favored regions, indicating a good quality model (>90%). Considering the unusuality measurement, G-factors were 0.35 (dihedrals), -0.23 (covalent), and 0.13 (overall), indicating that the model can be used for the proposed objective in this article, although some energy minimization may be needed for molecular simulation studies
